# Supplementary material for: Survey evaluation of dog owners’ feeding practices and dog bowls’ hygiene assessment in domestic settings
Source: PLoS One. 2022 Apr 6;17(4):e0259478. doi: 10.1371/journal.pone.0259478 (PMC8986020; doi:10.1371/journal.pone.0259478)
Supplement: Attachment — Submitted filename: Review Rebuttal.docx [file pone.0259478.s004.docx]

Emily Luisana, DVM

8805 Royalwood Court

Raleigh, NC 27613

[ektownse@gmail.com](mailto:ektownse@gmail.com)

(919) 418-4112

PLOS
1265 Battery Street, Suite 200
San Francisco, CA 94111
United States

January 6, 2022

Dear Reviewers,

Thank you for your time and consideration as well as your helpful feedback. I have addressed your comments, below, with our responses in bold with yellow highlight. The original data set has been included under ‘Supplemental Information’. Please do not hesitate to comment further with suggestions that may improve this paper.

Sincerely,

Emily Luisana, DVM

PONE-D-21-32996

Survey evaluation of dog owners’ feeding habits in a household setting and comparison of FDA hygiene protocols on dog bowl bacterial contamination as evaluated by total aerobic cell counts.

PLOS ONE

Upon re-submitting your revised manuscript, please upload your study’s minimal underlying data set as either Supporting Information files or to a stable, public repository and include the relevant URLs, DOIs, or accession numbers within your revised cover letter. For a list of acceptable repositories, please see <http://journals.plos.org/plosone/s/data-availability#loc-recommended-repositories>. Any potentially identifying patient information must be fully anonymized.

Important: If there are ethical or legal restrictions to sharing your data publicly, please explain these restrictions in detail. Please see our guidelines for more information on what we consider unacceptable restrictions to publicly sharing data: <http://journals.plos.org/plosone/s/data-availability#loc-unacceptable-data-access-restrictions>. Note that it is not acceptable for the authors to be the sole named individuals responsible for ensuring data access.

Reviewers' comments:

Reviewer's Responses to Questions

**Comments to the Author**

1. Is the manuscript technically sound, and do the data support the conclusions?

The manuscript must describe a technically sound piece of scientific research with data that supports the conclusions. Experiments must have been conducted rigorously, with appropriate controls, replication, and sample sizes. The conclusions must be drawn appropriately based on the data presented.

Reviewer #1: Partly

Reviewer #2: Yes

2. Has the statistical analysis been performed appropriately and rigorously?

Reviewer #1: No

Reviewer #2: Yes

3. Have the authors made all data underlying the findings in their manuscript fully available?

The [PLOS Data policy](http://www.plosone.org/static/policies.action#sharing) requires authors to make all data underlying the findings described in their manuscript fully available without restriction, with rare exception (please refer to the Data Availability Statement in the manuscript PDF file). The data should be provided as part of the manuscript or its supporting information, or deposited to a public repository. For example, in addition to summary statistics, the data points behind means, medians and variance measures should be available. If there are restrictions on publicly sharing data—e.g. participant privacy or use of data from a third party—those must be specified.

Reviewer #1: Yes

Reviewer #2: No

4. Is the manuscript presented in an intelligible fashion and written in standard English?

PLOS ONE does not copyedit accepted manuscripts, so the language in submitted articles must be clear, correct, and unambiguous. Any typographical or grammatical errors should be corrected at revision, so please note any specific errors here.

Reviewer #1: Yes

Reviewer #2: Yes

5. Review Comments to the Author

Please use the space provided to explain your answers to the questions above. You may also include additional comments for the author, including concerns about dual publication, research ethics, or publication ethics. (Please upload your review as an attachment if it exceeds 20,000 characters)

Reviewer #1: I can confirm that the subject matter of this study (Survey evaluation of dog owners’ feeding habits in a household setting and comparison of FDA hygiene protocols on dog bowl bacterial contamination as evaluated by total aerobic cell counts) is of interest and relevance for publication in PLOS ONE
Comments to the Authors:
- The authors did not present any hypothesis at the beginning
- add reference to statistical analysis
- correct temperature on the Celsius scale, the body weight - kg , add to weight ‘body’
In my opinion conclusion (summary) may be improved giving few key message/take home message to the readers. An idea may be to synthetize in 3-5 bullet the key results of the study, evidences and recommendation. This improvement will increase clearness and readability. Add a practical implications statement

**Thank you, this has been added/modified. A ‘reference to statistical analysis’ was presumed to be a citation for the software, which has been added. Please let us know if this more is desired.**

Reviewer #2: The present study provides interesting information about dog owner’s pet food and dish handling habits as well as, dog owner’s awareness of relevant FDA guidelines. Additionally, this work assesses the impact of specific FDA food handling and food bowl hygiene protocols on food bowl contamination, as determined by aerobic plate count. The survey findings indicated that the majority of participants were not aware of FDA guidelines and hence they do not follow the indicated food handling practices. On the other hand, the dog owners who were requested to follow specific guidelines during the study, presented low levels of compliance to the instructed protocols long-term. However, the implementation of these protocols resulted in significant reduction in aerobic plate count between pre- and post-bowl hygiene treatments, while the dish washing protocols seem to play important role in the microbial contamination reduction. Therefore, the authors emphasize the risk of bacterial contamination of the household for both humans and pets, as well as the need for better communication of the current guidelines and the development of more feasible recommendations for long-term compliance. Overall, this manuscript is well written and well structured. The reviewer has a few questions to be addressed and also a few suggestions to consider for improvement:

1. The microbial counts are usually expressed as colony-forming units rather than cells in plating methods, because more than one cell may be present on the same spot to give rise to a single colony. Furthermore, there are bacteria that grow in chains and a single colony may represent several cells. Therefore, it is more appropriate to use total aerobic count.

2. Total aerobic count may refer to all the aerobic microorganisms present in the samples. But, not all microorganisms are able to form colonies in a culture medium. Further to the above comment, the reviewer suggests Aerobic plate count (APC) or even Total plate count (TPC).

**Modified, thank you for the suggestion.**

3. It is mentioned in Lines 94-95 that ‘Owners were requested to complete one survey per dog with a maximum of two surveys per family’ and also in Lines 100-101 that ‘owners of 68 dogs (a total of 50 owners) were invited to complete a food bowl bacterial contamination study’. Why did the authors use survey data from the same dog owner, since it could be considered that the responses and their behaviour will be similar?

**Authors did not limit to one dog per family as our pilot study revealed that dogs within households may vary in food type, health and feeding behavior while having similar environments and owner compliance. This would allow for potential comparison of findings within and across households. Ultimately, sample size did not permit this type of analysis, although it could be expanded upon with future studies. Allowing two dogs per household also aided in increasing the overall sample size which improved accuracy. However, the authors elected to cap the study at 2 dogs per household to avoid overly leading the data.**

4. Line 134-135: Please describe the analysis in detail (which were the dependent and independent variables for the models). It is not so clear. In addition, Kruskall-Wallis can be used as an alternative to the Anova, when the assumption of normality or equality of variance is not met. Which assumption was not met? Where Kruskall-Wallis was used?

**Thank you, clarification has been added.**

5. Figure 3: y-axis label: APC or TPC (log10 CFU/cm2), x-axis label: Treatment group. The mean values are presented in this graph? Please, report also what the error bars represent.

**Graphs have been updated. Please let us know if further clarification is desired.**


6. The reviewer believes that the report of the 95% confidence intervals for the statistically significant results would be a significant addition.

**This has been added.**

Other comments:
• Line 62: this study cultured for? Please, rephrase
• Line 120: 10 cm2 environmental sampling template (Name of Company, City, Country)
• Line 122: (Name of Company, City, Country) for Butterfields solution
• Line 124: Petrifilm TM aerobic count plates (Name of Company, City, Country)
• Line 219: Please, report the method for multiple testing correction?
• Line 130: colony forming units per cm squared (CFU/cm2)
• Figures 1 and 2: axis labels
• Please, report microbial counts with only one digit after the decimal point. Given the accuracy, one digit is sufficient.

**All of the above have been added/modified, thank you. Please let us know if further clarification is desired.**

6. PLOS authors have the option to publish the peer review history of their article ([what does this mean?](https://journals.plos.org/plosone/s/editorial-and-peer-review-process#loc-peer-review-history)). If published, this will include your full peer review and any attached files.


If you choose “no”, your identity will remain anonymous but your review may still be made public.


**Do you want your identity to be public for this peer review?** For information about this choice, including consent withdrawal, please see our [Privacy Policy](https://www.plos.org/privacy-policy).

Reviewer #1: No

Reviewer #2: No

While revising your submission, please upload your figure files to the Preflight Analysis and Conversion Engine (PACE) digital diagnostic tool, <https://pacev2.apexcovantage.com/>. PACE helps ensure that figures meet PLOS requirements. To use PACE, you must first register as a user. Registration is free. Then, login and navigate to the UPLOAD tab, where you will find detailed instructions on how to use the tool. If you encounter any issues or have any questions when using PACE, please email PLOS at [figures@plos.org](mailto:figures@plos.org). Please note that Supporting Information files do not need this step.
