# Supplementary material for: Survey evaluation of dog owners’ feeding practices and dog bowls’ hygiene assessment in domestic settings
Source: PLoS One. 2022 Apr 6;17(4):e0259478. doi: 10.1371/journal.pone.0259478 (PMC8986020; doi:10.1371/journal.pone.0259478)
Supplement: Attachment — Submitted filename: Response to Reviewers 2.27.docx [file pone.0259478.s005.docx]

Emily Luisana, DVM

8805 Royalwood Court

Raleigh, NC 27613

[ektownse@gmail.com](mailto:ektownse@gmail.com)

(919) 418-4112

PLOS
1265 Battery Street, Suite 200
San Francisco, CA 94111
United States

February 28, 2022

Dear Reviewers,

Thank you for your time and consideration as well as your helpful feedback. I have instituted all suggestions with no questions outside of comments 4 and 39 with our reply in bold with yellow highlight. Please do not hesitate to comment further with suggestions that may improve this paper.

Sincerely,

Emily Luisana, DVM

ACADEMIC EDITOR:

Beyond the comment raised by the reviewer regarding the revised manuscript, there are some points that according to the Academic Editor’s view should also be addressed by the authors prior to its consideration for publication in PLOS ONE. These additional comments raised by the Academic Editor are listed below:

1. The title of the manuscript should be shorter and not so detailed; also, the period (.) at the end of the title should be deleted. Suggestion for title’s revision: “Survey evaluation of dog owners’ feeding practices and dog bowls’ hygiene assessment in domestic settings”. Corresponding revision for short title: “Evaluation of dog owners’ feeding and hygienic practices”.

2. L32: NCSU-IACUC and -IRB should be spelled out

3. L38, 39 and wherever else applicable: use past tense (e.g., had, stored, washed, prepared) when referring to results and protocols of the study.

4. L41 (and throughout the manuscript): mentioning different p-values may get confusing for the reader; please refer consistently to the p-value decided to be treated as significance level in this study (P<0.05 for significant difference and P≥0.05 for non-significant differences) throughout the manuscript; there is no need for the specific attained p-value to be mentioned.

**Changed as recommended, values p<0.01 or <0.001 were rounded as well.**

5.  L45: there is no such thing as “high-risk” households but “high-risk populations” or “high-risk individuals”

6. L49-50: this sentence should be revised for clarity

7. L55: the phrase “the actual act of feeding” sounds weird…please revise to the simpler phrase “Pet feeding involves an interplay among the pet, the owner and the feed”.

8. L65 and 66: correct to “Staphylococcus spp.” (“spp.” should not be italicized), “pseudomonads” (this is not a genus name but a bacterial group name and thus, it should not be italicized) and “Enterobacteriaceae” (family names should not be italicized).

9. L75: revise to the “U.S. Food and Drug Administration”

10. L81: use “implementation” in the place of “institution”

11. L87, 101, 147 etc.: please delete the dash (-) after each subsection’s title

12. L110-115: the sentence “Treatment group…wash and dry cycle” is rather long and inevitably complicated; please consider revising by splitting to at least two distinct sentences”

13. L137: the phrase “due to percent change from 0 not being defined” could be omitted.

14. L138-140: which linear models did you try?

15. L156: please add a comma (,) prior to “whereas”

16. L157: please mention the “less than 2% each” in parentheses comprehensively for the corresponding illnesses.

17. L165: the “>25%” is not useful information, particularly when someone reads a phrase like “Lower levels of compliance”…either refer to a “<…” statement or provide a range for the corresponding values to give the reader a better idea of the magnitude

18. Table 1: The "yes" or "no" statement is confusing when referring to compliance, where someone only "yes” would expect to be pertinent.

19. Table 2: The presentation format could be improved with presenting first the answer, followed by the percentage in parentheses or after “:”

20. L183-185: There is no need to repeat information in the text of the manuscript already provided in a tabular format (Table 2 in this particular case). Similarly in L190-192 and in L195.

21. L199: please add a comma (,) prior to “respectively”

22. L201: please revise the phrase “<1% and 3%, APC and overall, respectively” for clarity

23. L206 and wherever else applicable: use “h” instead of hours” and “min” instead of “minutes”

24. L209: change “between” to “among”

25. L210: please add a period after “assessment”

26. L217: please correct to “data were…”; “data” is the plural form of “datum”

27. L219: please delete the period (.) after “APC”

28. L225: the “non-significant difference” should be denoted by “p≥0.05” (and not by p<0.05); please correct accordingly

29. L235: which linear regression model did you use

30. L241: use directly the already spelled out “APC”

31. L246: change “Whereas” to “On the other hand”

32. L251: which statistical model do you refer to?

33. L259: correct to “…and did not follow”

34. L272: revise to “According to Weese et al. (2010), dog bowls were 17 times more likely…”

35. L282: please use “efficacy of sanitation” instead of “degree of sanitation”

36. L292: raw meat cannot be infected but contaminated

37. L294-296: the sentence “The effects of cross-contamination may extend beyond bacterial contamination when one considers that 9% of pet owners reported adding medications or supplements into their pets’ food bowls.” is not clear; please revise.

38. L299: “…may have implications as far as increased microbial risk”: it is not clear what you mean to say here

39. L307: veterinarians do not have consumers…most likely, “clients” is a more appropriate word here

Added both clients and consumers as veterinarians have clients and pet food manufacturers and retailers have customers/consumers. Please let me know if you find this unacceptable.

40. L309: use “allow for” instead of “institute”

41. L315: microbial growth was not assessed in the context of this study

42. L320: use “establishment” or “development” or “implementation” instead of “institution”.

**Comments to the Author**

6. Review Comments to the Author

Please use the space provided to explain your answers to the questions above. You may also include additional comments for the author, including concerns about dual publication, research ethics, or publication ethics. (Please upload your review as an attachment if it exceeds 20,000 characters)

Reviewer #2: Please, check again the y-axis label for Figure 3. The correct is APC (log CFU/cm^2). Also add in the figure legend that the results were expressed as mean (± standard error of the mean, SEM) log CFU/cm^2.
